# A LS-SVM based Measurement Points Classification Algorithm for Adjacent Targets in WSNs

**DOI:** 10.3390/s19245555

**Published:** 2019-12-16

**Authors:** Xiang Wang, Zong-Min Zhao, Tao Wang, Zhun Zhang, Qiang Hao, Xiao-Ying Li

**Affiliations:** 1 School of Electronic and Information Engineering, Beihang University, Beijing 100191, China; wxiang@buaa.edu.cn (X.W.); wt860122@buaa.edu.cn (T.W.); microzhun@buaa.edu.cn (Z.Z.); haoqiang1994@buaa.edu.cn (Q.H.); 2 Institute of Medical Information, Chinese Academy of Medical Sciences, Beijing 100020, China; lixiaoying@imicams.ac.cn

**Keywords:** wireless sensor networks (WSNs), measurement origin uncertainty, localization and tracking, least square support vector machine (LS-SVM)

## Abstract

In wireless sensor networks (WSNs), the problem of measurement origin uncertainty for observed data has a significant impact on the precision of multi-target tracking. In this paper, a novel algorithm based on least squares support vector machine (LS-SVM) is proposed to classify measurement points for adjacent targets. Extended Kalman filter (EKF) algorithm is firstly adopted to compute the predicted classification line for each sampling period, which will be used to classify sampling points and calculate observed centers of closely moving targets. Then LS-SVM algorithm is utilized to train the classified points and get the best classification line, which will then be the reference classification line for the next sampling period. Finally, the locations of the targets will be precisely estimated by using observed centers based on EKF. A series of simulations validate the feasibility and accuracy of the new algorithm, while the experimental results verify the efficiency and effectiveness of the proposal.

## 1. Introduction

Nowadays, with the development of precision sensors to monitor physical or environmental conditions, the applications of WSNs have been motivated by various industrial and consumer systems, as well as military usages [1,2,3]. Among the increasing research and applications of WSNs, an important field is target localization and tracking [4,5]. Generally, the location problem in WSNs falls into two categories: range-free or range-based method [6]. While the former, depending on a kind of communication device (such as ZigBee, WiFi, Bluetooth, RF, and so on), has exhibited the advantage of easy implementation without additional ranging sensors, the latter, tracking targets based on the distance measurement sensors and angle sensor, demonstrates much better positioning accuracy. In this paper, we will mainly focus on the range-based localization method.

Broadly speaking, the traditional methods of localization and tracking contain Kalman filter (KF), extended-KF (EKF), Unscented-KF and so on [7,8,9]. The basic idea of these algorithms is to construct suitable state equations and predict the location of targets for the next moment. Meanwhile, the observed values will also be obtained from sensor systems, which are then utilized to estimate target positions combined with predicted values. These algorithms are well suited to the case where one object corresponds to one measurement per scan. However, in WSNs, it is quite common for one target to generate multiple measurements, which is generally called extended target. There are many reasons for this phenomenon, such as the improvement of sensor resolution, the target being close to the sensor, and multiple sensors working together. In WSNs, there exist some special features for acquisition of observation values, which is a problem for the measurement of uncertain origin [10]. Although multiple sensors have been applied to observe several targets in WSNs, it is still not clear whether a measurement point belongs to a specific target or not, especially for adjacent targets. Obviously, in order to get precise observation and estimation value for targets, it is a major requisite to accurately classify these sampling points.

In general, there are two kinds of state-of-the-art methods dealing with sampling points for respective targets: with or without the probability model [11,12,13,14,15]. While the computational complexity of the former increases as the model becomes more complicated, the latter saves a large number of complex calculations and includes two different methods: clustering and classification algorithms. Clustering is a process of unsupervised machine learning without the training of sample data, and group target data set by designing a suitable distance metric [16,17]. Besides, the clustering algorithm is fairly effective and suitable for acquiring observations in WSNs. However, in cases where targets move closely and their sampling points overlap each other, the performance of the clustering algorithm would dramatically decrease due to different distributions of these overlapped points. In contrast, the classification algorithms have favorable stability, and the most representative one among them is SVM algorithm. As SVM avoids the traditional process from generalization to deduction, it largely simplifies the usual classification problem. Especially, LS-SVM optimizes the algorithm solution and improves computing efficiency [18,19]. Nevertheless, being a supervised machine learning algorithm, LS-SVM trains the sample data before classification and has been rarely used in applications with dynamic systems, such as tracking problems in WSNs. In this paper, a novel maneuvering targets classification algorithm is developed by combining LS-SVM and EKF to classify sampling points, which are then used to calculate the observed centers for targets and evaluate the positions of targets based on the EKF algorithm.

The remainder of this paper is organized as follows. Section 2 mainly describes the related works. In Section 3, we describe the principles of the proposed algorithm in detail. Section 4 is about the simulation that verifies the classification and tracking performance, followed by the experiment and discussion about the proposal in Section 5. At last, Section 6 concludes this paper.

## 2. Related Work

Recently, with the improvement of sensor resolution, the problem of extended target recognition and positioning has attracted a lot of research attention. One of the key issues is how to identify two adjacent extended targets and locate them separately.

Maximum Likelihood Probabilistic Multi-Hypothesis Tracker (ML-PMHT) can reliably discriminate between the target and clutter, based on the calculation of peak point’s PDF in ML-PMHT log-likelihood ratio (LLR) due to clutter and also the peak point’s Probability Density Function (PDF) in LLR due to the target. In order to track the original target from measurement points generated by both the original target and interfering target, Steven Schoenecker replaced the clutter with a second, independently parameterizable target, on the basis of ML-PMHT [20]. However, sometimes the peak of the LLR surface that it generates can merge with the original target peak, and the ability to track the original target is limited, especially when an interfering target is strong enough or close enough. Based on previous work, [21] developed a statistical method to watch how close two targets were to each other, and the system still had the ability to recognize them, by checking whether the LLR peaks of the two targets coincided. Li proposed an improved partitioning algorithm for a Gaussian inverse Wishart probability hypothesis density (GIW-PHD) filter to solve the problem where the sub-partitioning algorithm failed to handle cases where targets were of different sizes, and the Mahalanobis distances was employed to distinguish among measurement cells of different sizes for extended targets [22]. However, this approach seems to not be sensitive to either differences in target size or target maneuvering.

As the applications of extended targets quickly increase, the research on target location is gradually developed for the method based on clustering. The probability-based method is used to classify each measurement point, and the great number of equivalent measurement points will lead to very large computational complexity. For this issue, adaptive weight Fuzzy C-Means clustering (AWFCM) algorithm was used to track multi-targets under the cross tracks situation [23], where a distance in new metric space was defined to restrain the error range of data association clustering centers for samples with noise points and cross tracks. Besides, the improved weights dependent on dots’ density were introduced to reduce the negative effect of imbalanced data sets. Another method of Clustering-Based Multiple Hypothesis Multi-target Localization was presented for the same purpose, which divided a large number of hypotheses into several independent clusters, as well as hypothesis update, hypothesis pruning and global hypothesis generation were completed independently in each cluster [24]. Additionally, a clustering method-based Evolutionary Kernel Fuzzy C-Means clustering (EKFCM) algorithm was adopted to identify and locate targets oriented to multi-target tracking in wireless sensor networks [13], which firstly applied the clustering number recognition algorithm to filter out outliers and then calculate initial cluster centers based on the density of each measurement data. At last, the density factors of these measurement data were fused into the Gaussian kernel function to improve the accuracy of the cluster center, and the accurate position of each target at the current moment was calculated according to the predictive position and measurement data set of the corresponding cluster.

Relatively speaking, the clustering algorithm could quickly and effectively classify the measurement points and track the measurement points belonging to each extended target, so as to solve the problem of measurement origin uncertainty. The accuracy of the clustering algorithm to classification of measurement points will directly affect the accuracy of the target location. However, when two objects are close to each other, the overlapping phenomenon of measurement points will result in a classification error of the clustering algorithm and affect the positioning accuracy. In order to solve this problem, we use the classification method based on LSSVM to classify the measurement points for adjacent targets, which will further improve the accuracy of the target location.

## 3. The Proposed Algorithm

In this section, we will describe the proposed algorithm in detail.

### 3.1. Algorithm Overview

**Figure 1:**
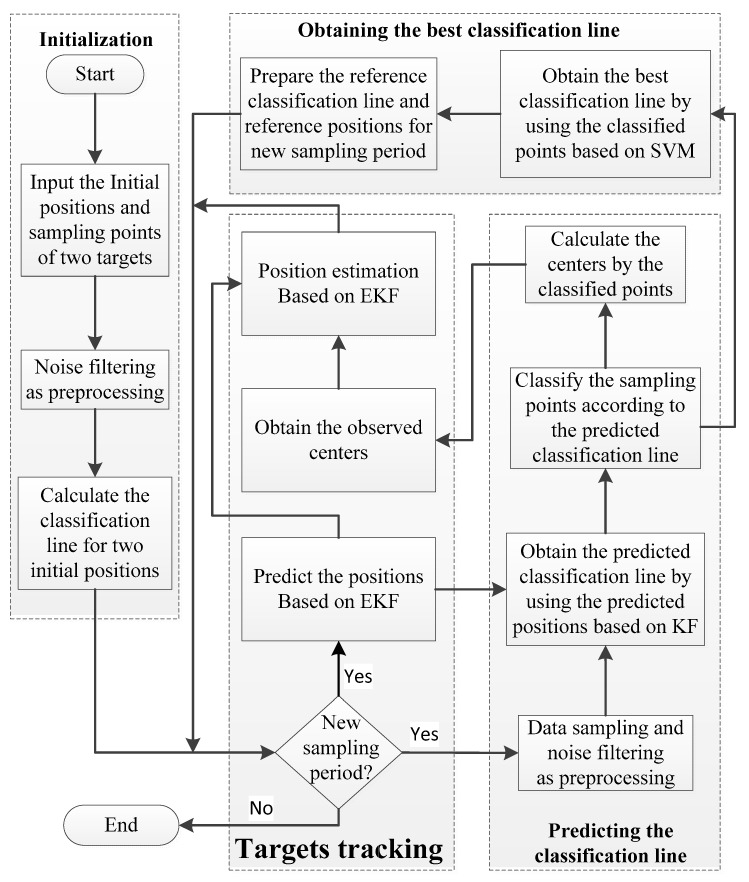
Flowchart of the proposed algorithm.

As shown in Figure 1, the whole process of our new algorithm consists of four portions: initialization, predicting the classification line, obtaining the best classification line, and target tracking. Initialization provides the initial values for EKF and the original sampling points for LS-SVM algorithm, as well as the best classification line. The predicted positions of targets will be calculated based on the EKF algorithm and then applied to predict the classification line. Besides, the measurement points sampled by sensors in WSNs with noise points filtered out by using some simple filter would be classified by the predicted classification line. Subsequently, the geometric centers of these classified points will be regarded as observed centers of targets, which could be adopted to estimate the positions of targets based on EKF algorithm. Meanwhile, the classified points will also be devoted to train the best classification line based on LS-SVM algorithm, which will be taken as the reference classification line for the next sampling period.

Here, to facilitate the description of the algorithm, we make a few points in advance. Firstly, the classification line is used hereinafter to replace the classification surface, as it is a straight line in a 2-D area. Secondly, because the sampling points always contain valid measurement points of targets and also invalid noise points, we will filter out the noise points far from targets based on the density of the sampling points. Thus, the sampling points can be considered as valid measurement points after filtering. Finally, since it is the most common case that two targets move closely compared with more targets, we will take two targets as the example to show the principle of the proposed algorithm.

### 3.2. EKF algorithm for Target Tracking

In order to guarantee the algorithm works well under nonlinear systems, EKF algorithm is utilized to track targets. Firstly, we build a state model for each target:

(1) $$Z\left(k\right)={\left[x\left(k\right)\;\dot{x}\left(k\right)\;y\left(k\right)\;\dot{y}\left(k\right)\right]}^{T}$$

where x(k) and y(k) represent the x-axis and y-axis positional coordinates of the target at time k respectively, $\dot{x}(k)$ and $\dot{y}(k)$ indicate the speed of the target along the x-axis and y-axis directions at moment k. According to EKF, the state equation of the target is:

(2) Z(k+1)=Φ(k+1|k)Z(k)+G(k)W(k)+ϕ(k)

where Φ(k+1|k) represents the state transition matrix, and G(k) and W(k) denote the noise driving matrix and input white noise. Here, ϕ is a nonrandom term. Besides, the initial value is set as:

(3) Z(0)=E[Z(0)]

The prediction equation is:

(4) $$\overset{⌢}{Z}\left(k+1|k\right)=f\left(\overset{⌢}{Z}\left(k|k\right)\right)$$

and the prediction covariance matrix is:

(5) $$P\left(k+1|k\right)=\Phi \left(k+1|k\right)P\left(k|k\right)\Phi^{T}\left(k+1|k\right)+Q\left(k+1\right)$$

where Q is the variance of W, and the initial value of the prediction covariance is:

(6) P(0)=var[Z(0)]

The predicted positions of targets will be adopted to predict the classification line, and the observed centers O(k+1) of targets will be obtained from Section 3.3. The estimation equation is:

(7) $$\overset{⌢}{Z}\left(k+1|k+1\right)=\overset{⌢}{Z}\left(k+1|k\right)+K\left(k+1\right)\left[O\left(k+1\right)-h\overset{⌢}{Z}\left(k+1|k\right)\right]$$

(8) $$K\left(k+1\right)=P\left(k+1|k\right)H^{T}\left(k+1\right){\left[H\left(k+1\right)P\left(k+1|k\right)H^{T}\left(k+1\right)+R\left(k+1\right)\right]}^{-1}$$

(9) P(k+1)=[I−K(k+1)H(k+1)]P(k+1|k)

(10) $$H\left(k\right)=\frac{\partial h}{\partial \overset{⌢}{X}\left(k\right)}|{}_{X\left(k\right)=\overset{⌢}{X}\left(k\right)}$$

where h denotes the observation matrix.

### 3.3. Predicting the Classification Line

**Figure 2:**
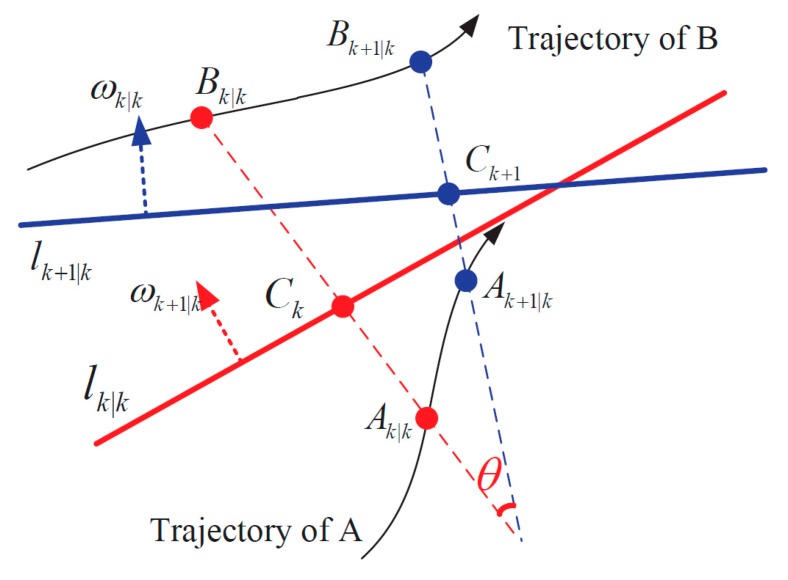
Transformation for the classification line.

In this subsection, we use the predicted positions of targets from Section 3.2 to predict the classification line, which will be devoted to classify sampling points, calculate observed centers, and also estimate positions of targets. Figure 2 shows the geometric relationship between the best classification line at time k and the predicted classification line at moment k+1. Here, $A_{k|k}$ and $B_{k|k}$ are the geometric centers of classified points, and $l_{k|k}$ is the best classification line at time k, which is also the reference classification line at k+1. $C_{k}$ is the intersection point between $l_{k|k}$ and connection line $A_{k|k}B_{k|k}$. Similarly, $A_{k+1|k}$ and $B_{k+1|k}$ are the predicted positions of targets at time k+1. $l_{k+1|k}$ is the predicted classification line to be calculated below, with $C_{k+1}$ the intersection point between $A_{k+1|k}B_{k+1|k}$ and $l_{k+1|k}$. The specific process holds six steps.

**Step 1:** Input the initial parameters. 

The initial parameters mainly refer to the best classification line and observed centers at the very beginning of the algorithm. For the following sampling periods, the best classification line at time k will be regarded as the reference classification line at moment k+1. Later, after predicting targets’ positions at time k+1 based on EKF, the predicted classification line at time k+1 will be obtained by transforming the reference classification line according to the positional relationship between predicted targets positions at time k+1 and observed targets centers at k(through following steps 2–5).

**Step 2:** Calculate the intersection point $C_{k}$.

The intersection point $C_{k}(x_{k|k}^{\left(C\right)},y_{k|k}^{\left(C\right)})$ could be calculated by solving the straight line equations below:

(11) $$\omega_{k|k}\cdot {\left[x_{k|k},y_{k|k}\right]}^{T}+b_{k|k}=0$$

(12) $$y_{k|k}=y_{k|k}^{\left(A\right)}+\left[\frac{y_{k|k}^{\left(B\right)}-y_{k|k}^{\left(A\right)}}{x_{k|k}^{\left(B\right)}-x_{k|k}^{\left(A\right)}}\right]\left[x_{k|k}-x_{k|k}^{\left(A\right)}\right]$$

where (11) is the equation of the best classification line $l_{k|k}$ with normal vector $\omega_{k|k}$, and (12) is the equation of line $A_{k|k}B_{k|k}$. We would also compute the scaling factor $\alpha_{s}$, which denotes the proportion of point $C_{k}$ between $A_{k|k}$ and $B_{k|k}$.

(13) $$\alpha_{s}=\frac{y_{k|k}^{\left(C\right)}-y_{k|k}^{\left(B\right)}}{y_{k|k}^{\left(A\right)}-y_{k|k}^{\left(B\right)}}$$

**Step 3:** Compute the predicted intersection point $C_{k+1}$.

Assume that the relative position of predicted classification line $l_{k+1|k}$ between two targets is persistent for two adjacent moments. Thus, the scaling factor $\alpha_{s}$ for time k+1 will be equal to the one at moment k. According to predicted positions of targets $A_{k+1|k}$ and $B_{k+1|k}$, we will calculate the predicted intersection point $C_{k+1}(x_{k+1|k}^{\left(C\right)},y_{k+1|k}^{\left(C\right)})$:

(14) $$\left[\begin{array}{l} x_{k+1|k}^{\left(C\right)}\\ y_{k+1|k}^{\left(C\right)} \end{array}\right]=\alpha_{s}\left[\begin{array}{l} x_{k+1|k}^{\left(A\right)}\\ y_{k+1|k}^{\left(A\right)} \end{array}\right]+\left(1-\alpha_{s}\right)\left[\begin{array}{l} x_{k+1|k}^{\left(B\right)}\\ y_{k+1|k}^{\left(B\right)} \end{array}\right]$$

**Step 4:** Calculate the normal vector of $l_{k+1|k}$.

The predicted classification line $l_{k+1|k}$ would be transformed from the best classification line $l_{k|k}$ at time k. Thus, the normal vector of $l_{k+1|k}$ could be obtained by getting the rotation angle θ of two lines $AB_{k|k}$ and $AB_{k+1|k}$, which equals to the rotation angle between the normal vector of $l_{k|k}$ and normal vector of $l_{k+1|k}$.

(15) $$\cos\theta =\frac{\vec{AB_{k|k}}\cdot \vec{AB_{k+1|k}}}{\left\|\vec{AB_{k|k}}\right\|\left\|\vec{AB_{k+1|k}}\right\|}$$

(16) $$\sin\theta =\frac{\vec{AB_{k|k}}\times \vec{AB_{k+1|k}}}{\left\|\vec{AB_{k|k}}\right\|\left\|\vec{AB_{k+1|k}}\right\|}$$

here $\vec{AB_{k|k}}$ denotes the vector of point $A_{k|k}$ to point $B_{k|k}$, and $\vec{AB_{k+1|k}}$ presents the vector of point $A_{k+1|k}$ to point $B_{k+1|k}$ respectively. Then, the normal vector of $l_{k+1|k}$ is:

(17) $$\omega_{k+1|k}=\omega_{k|k}\left[\begin{array}{cc} \cos\theta & \sin\theta \\ -\sin\theta & \cos\theta \end{array}\right]$$

**Step 5:** Obtain the predicted classification line.

The predicted classification line *l*_k+1|k_ can be described as below:

(18) $$\omega_{k+1|k}\cdot {\left[x_{k+1|k},y_{k+1|k}\right]}^{T}+b_{k+1|k}=0$$

where $b_{k+1|k}$ will be derived by taking point $C_{k+1}(x_{k+1|k}^{\left(C\right)},y_{k+1|k}^{\left(C\right)})$ into (18).

**Step 6:** Calculate the observed centers of targets.

After obtaining the predicted classification line, each sampling point is put into the following formula for classification.

(19) $$M(X_{i})=sgn(\omega_{k+1|k}\cdot {\left[x_{k+1,i},y_{k+1,i}\right]}^{T}+b_{k+1|k})$$

The sampling points will be classified into two groups, the geometric center of each group is supposed to be the observed center for each target, which could be adopted to estimate the positions of targets based on EKF (see Section 3.2).

The method for predicting the classification line is presented in Algorithm 1 below.

**Algorithm 1** Predicting the classification lineInput: best classification line and observed centers of targets at time k; Output: predicted classification line and observed centers of targets at moment k+1; 1: Input the best classification line and observed centers of targets at time k as initial  parameters, get the reference classification line and predicted targets positions $A_{k+1|k}$
 and $B_{k+1|k}$ at moment k+1; 2: Calculate the intersection point $C_{k}$ and scaling factor $\alpha_{s}$ according to the straight line  equations; 3: Compute the predicted intersection point $C_{k+1}$ on the basis of scaling factor $\alpha_{s}$ and  predicted positions of targets $A_{k+1|k}$,$B_{k+1|k}$; 4: Calculate the normal vector of $l_{k+1|k}$ based on positions of targets at time k and k+1; 5: Obtain the predicted classification line at moment k+1 with intersection point $C_{k+1}$ and  normal vector of $l_{k+1|k}$; 6: Calculate the observed centers of targets at time k+1 after classifying the sampling points  with the predicted classification line, which will be utilized to track targets based on EKF.

### 3.4. Training the Best Classification Line

By using the predicted classification line to classify the sampling points, we could effectively find the observed centers of targets at each time. However, the predicted classification line obtained at time k may sometimes bring some classification errors for next moment k+1. Subsequently, the following classification is affected by the accumulated error before, namely misclassification. In order to reduce the impact of the accumulated error, before the closing algorithm for the current sampling period k and beginning of the new period k+1, we will retrain the data set and get the best classification line of classified points, which will be regarded as the reference classification line for the next sampling period k+1. In order to reduce computational complexity, we use the LS-SVM to train the best classification line. The LS-SVM turns the inequality constraints of the original SVM algorithm into equality constraints, thus the solving quadratic programming is replaced by solving linear equations [18]. According to the classified sampling point set ${\left\{x_{i},y_{i},z_{i}\right\}}_{i=1}^{l}$ with input data $\left\{x_{i},y_{i}\right\}$ and corresponding binary class labels $z_{i}\in \{-1,+1\}$, the LS-SVM classifier is obtained by reformulating the minimization problem as:

(20) $$\underset{\omega }{\min}\Phi (\omega ,e)=\frac{1}{2}(\omega \cdot \omega )+\gamma \frac{1}{2}\sum_{i=1}^{l}e_{i}^{2}$$

subject to

(21) $$z_{i}(\omega \cdot {\left[x_{i},y_{i}\right]}^{T}+b)=1-e_{i},i=1,\ldots ,l$$

where $e_{i}$ denotes the slack variable, γ presents the weight to balance best hyperplane and minimum deviation, and · presents the scalar product of two vectors. Subsequently, the Lagrangian function is constructed as:

(22) $$L\left(\omega ,b,e,\alpha \right)=\Phi (\omega ,e)-\sum_{i=1}^{l}\alpha_{i}\left[z_{i}\left(\omega \cdot {\left[x_{i},y_{i}\right]}^{T}+b\right)-1+e_{i}\right]$$

where $\alpha_{i}\in R$ denote the Lagrange multipliers. The conditions for optimality are

(23) $$\{\begin{array}{l} \frac{\partial L}{\partial \omega }=0\to \omega =\sum_{i=1}^{l}\alpha_{i}\left[x_{i},y_{i}\right],\\ \frac{\partial L}{\partial b}=0\to \sum_{i=1}^{l}\alpha_{i}=0,\\ \frac{\partial L}{\partial e_{i}}=0\to \alpha_{i}=\gamma e_{i},i=1,\ldots ,l,\\ \frac{\partial L}{\partial \alpha_{i}}=0\to z_{i}\left(\omega \cdot {\left[x_{i},y_{i}\right]}^{T}+b\right)+e_{i}-1=0,i=1,\ldots ,l. \end{array}$$

By solving the linear Equations (23), we can get the best classification line. The method for training the best classificiation line is shown in Algorithm 2.

**Algorithm 2** Training the best classification lineInput: predicted classification line and observed measure point set ${\left\{x_{i},y_{i}\right\}}_{i=1}^{l}$ at time k+1; Output: best classification line at moment k+1; 1: Input the predicted classification line and observed measure point set  ${\left\{x_{i},y_{i}\right\}}_{i=1}^{l}$ at time k+1; 2: Classify the observed measure point set ${\left\{x_{i},y_{i}\right\}}_{i=1}^{l}$ by using the predicted  classification line according to Equation (19), which will get the classified  sampling point set ${\left\{x_{i},y_{i},z_{i}\right\}}_{i=1}^{l}$ with the binary class labels $z_{i}\in \{-1,+1\}$; 3: Substituting ${\left\{x_{i},y_{i},z_{i}\right\}}_{i=1}^{l}$ into Equation (23) to compute the parameter  ω and b, and finally get the best classification line at moment k+1.

## 4. Algorithm Simulation and Validation

In this section, the classification and tracking performance of the presented method will be simulated for algorithm validation.

### 4.1. Parameter Selection

**Figure 3:**
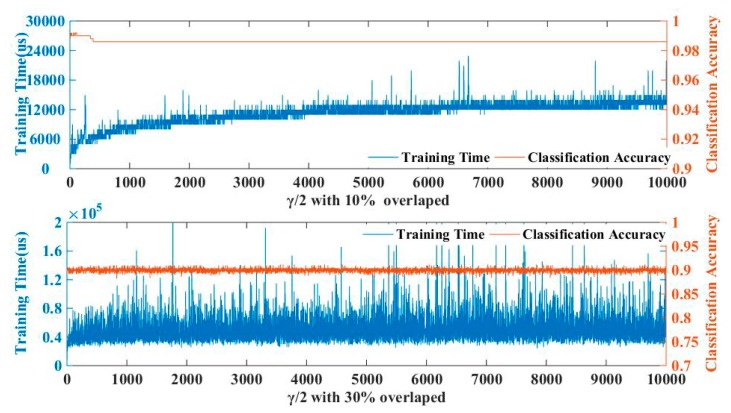
Parameter Selection for γ.

The parameter γ, which is shown in (20), is needed to be determined before program running. Theoretically, a bigger γ will increase the difficulty of calculation, while a smaller one will reduce the balance effect. In order to determine a proper γ, we simulated a classification by using the measurement points of two targets in different overlapping ranges and optimized the value of γ/2 from 1 to 10000. The overlap range is set from 0% to 30% for simulation, with the simulation result listing in Figure 3. The subfigure above shows the influence of the selection of γ on the running time and classification accuracy under 10% of the overlapped range, and the subfigure below lists the test under 30% of the overlapped range. Clearly, we get a balance between running time and classification accuracy when the γ/2 will be assigned within 1000. 

### 4.2. Simulation of Classification Performance

**Figure 4:**
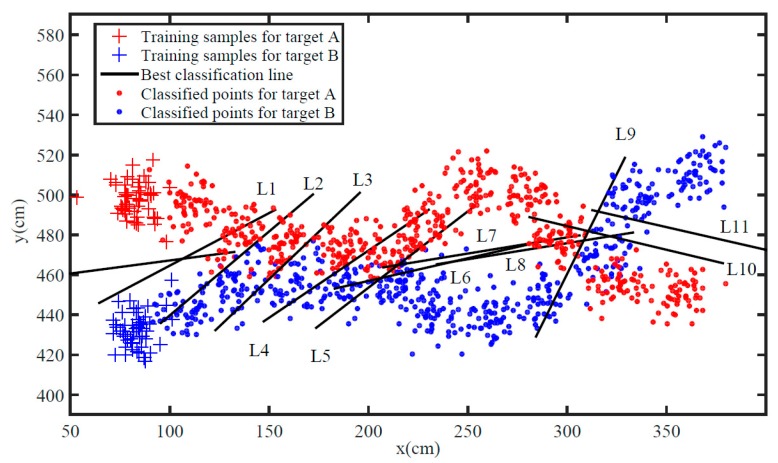
The classification process.

In order to verify the classification performance of the proposed algorithm, we firstly simulate the classification of the two targets within 11 groups of continuous sampling periods, which is shown in Figure 4. Here, L represents the best classification line for each sampling period. The whole simulation process involves two different motion states of targets: parallel state (L2–L5) and crossing state (L9–L10). Figure 4 reveals that the proposed algorithm can basically distinguish the sampling points of two targets, even though two targets are sometimes very close to each other. Besides, once two targets move separately (L6–L8, L11), the best classification line will effectively reduce accumulative error.

**Figure 5:**
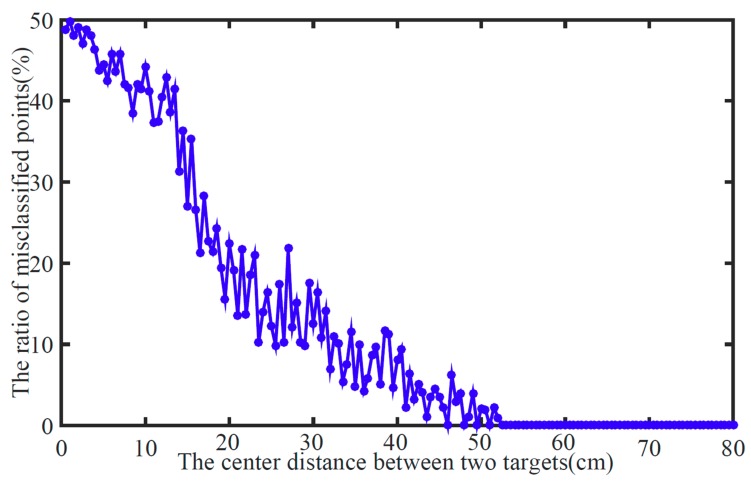
The misclassification probability.

Moreover, a Monte Carlo simulation with 5000 s was taken to analyze the classification performance. We have recorded the number of misclassified points and center distance between two targets. Figure 5 reports the probabilities of misclassification over center distances of two targets. As the radius of targets is set to 20 cm, the classification performance of our proposed algorithm becomes increasingly promising while the center distance is bigger than 30 cm, especially 50 cm (while two targets completely separate from each other).

### 4.3. Simulation of Tracking Performance

In order to evaluate the performance of the proposed algorithm for tracking adjacent targets, several simulations have been done and compared with some existing algorithms, namely K-means [16], Fuzzy C-means(FCM) [14] and EKFCM [13] clustering algorithm, which are all combined with the same tracking EKF algorithm. Besides, the root mean square error (RMSE) and Mean RMSE (MMSE) of the position are chosen as performance metrics [25].

**Figure 6:**
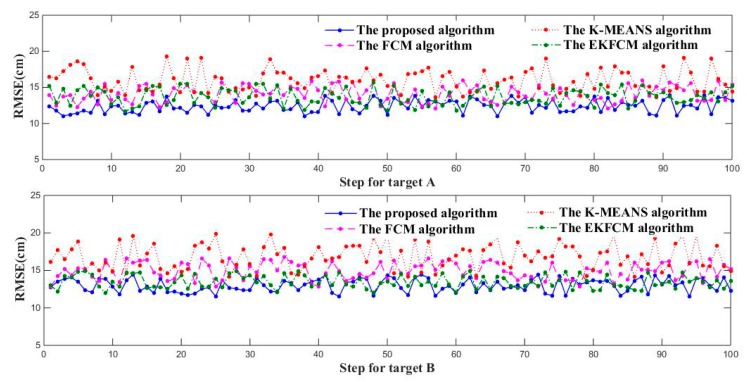
RMSEs across 100 Monte Carlo simulations.

The RMSE and MMSE of position are respectively defined as follows:

(24) $$RMSE=\sqrt{\frac{1}{T}\sum_{k=1}^{T}((x_{k}^{s}-{\hat{x}}_{k}^{s}{)}^{2}+{\left(y_{k}^{s}-{\hat{y}}_{k}^{s}\right)}^{2})}$$

(25) $$MMSE=\sqrt{\frac{1}{MT}\sum_{s=1}^{M}\sum_{k=1}^{T}((x_{k}^{s}-{\hat{x}}_{k}^{s}{)}^{2}+{\left(y_{k}^{s}-{\hat{y}}_{k}^{s}\right)}^{2})}$$

where $\left(x_{k}^{s},y_{k}^{s}\right)$ and $\left({\hat{x}}_{k}^{s},{\hat{y}}_{k}^{s}\right)$ represent true and estimated positions at time k of the s-th Monte Carlo simulation, and M = 100 and T = 1000 s are the total number of Monte Carlo runs and simulation time separately. Figure 6 shows the RMSEs of K-means, FCM, EKFCM and the proposed algorithm, with Table 1 illustrating their MMSE. It is obvious that the RMSE of the proposed algorithm is almost smaller than the ones of K-means, FCM and EKFCM algorithms in each Monte Carlo run, and the MMSE of our proposal is reduced at least 20% and 10% compared with K-means and FCM algorithm. Compared with EKFCM algorithm, the MMSE of the proposed method is reduced almost 10%. Besides, we calculated the average running time of each algorithm shown in Table 1, where the proposed method takes a little longer time to solve SVM equations rather than the addition-based clustering algorithm. Therefore, in terms of feasibility and accuracy of tracking adjacent targets, the provided algorithm will be more suitable than K-means, FCM and EKFCM.

## 5. Experimental Evaluation

Besides algorithm simulation, a series of experiments are conducted to validate the efficiency and effectiveness of our proposal. In this section, we firstly introduce the implementation of experiments and then analyze and discuss experimental results.

### 5.1. Experimental Implementation

**Figure 7:**
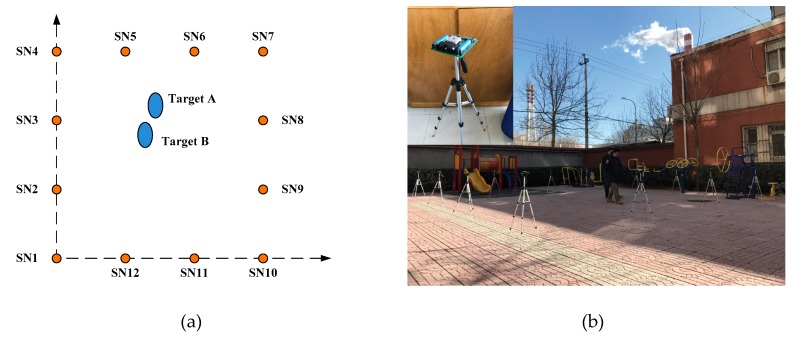
Experimental setup: the distribution of the sensor. (**a**) The distribution of sensors; (**b**) The experimental scene.

A WSN platform with 12 sensor nodes was set up (Figure 7) covering a surveillance area of 7 by 7 square meters. The positional coordinate for sensor node is SN1 (0,0), SN2 (0,2.33), SN3 (0,4.67), SN4 (0,7), SN5 (2.33,7), SN6 (4.67,7), SN7 (7,7), SN8 (7,4.67), SN9 (7,2.33), SN10 (7,0), SN11 (4.67,0), and SN12 (2.33,0) in meters. Vertically, the sensor nodes are all placed at the height of 1.15 m.

**Figure 8:**
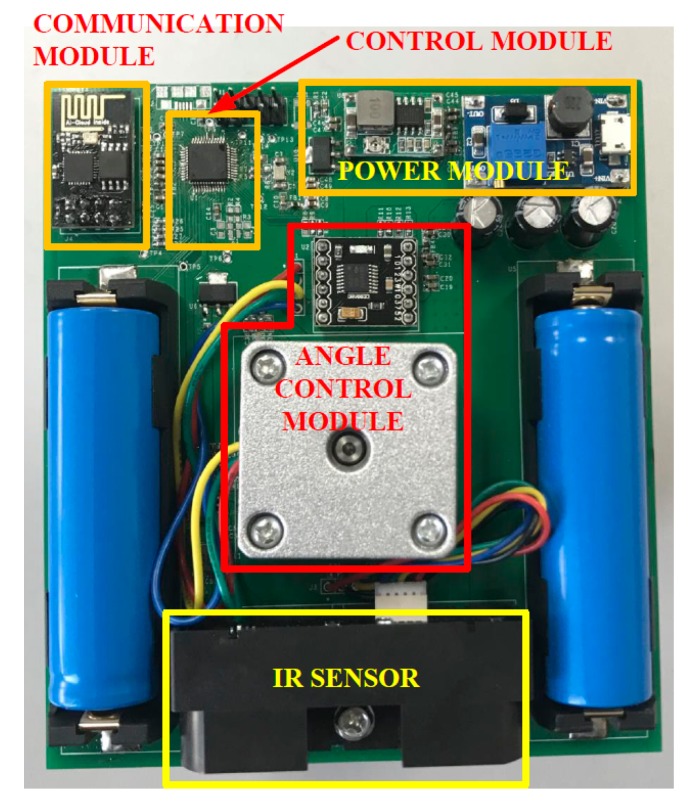
The details of the sensor.

The sensor node consists of five parts: infrared ranging (IR) sensor, angle control module, wireless communication module, control module, and power supply module, which is shown in Figure 8. Specifically, the effective detection distance of the IR sensor is up to 5 meters, and the angle control module drives a stepping motor with a step angle of 7.2°. Here, a sampling period is defined as the time required by the sensor node to turn half a circle. Each sampling period is 0.5 s and includes 25 sampling windows. All the sampling data will be sent to the host computer by wireless communication module with model ESP8266. The control module here is hired to initialize and manage other modules. Two people are the targets of the practical experiment. Once beginning, they enter the surveillance region with a speed of approximately 1m/s and walk along the predefined routes. During the whole experiment, they will move closely with each other and even cross several times. While two persons keep walking, the sensor nodes sample target positions and then update the data to the host computer, which will be analyzed by the proposed algorithm to track adjacent targets.

Besides, in order to compare the tracking efficiency of these algorithms, it is necessary to obtain true positions of each target. Therefore, a kind of device for recording time and imprinting on the ground is installed on the shoes of every person. When people walk, a mark will be printed on the ground for each footstep as well as the exact time will be recorded by the device. After the experiment is completed, all the marks will be detected and the center between the two adjacent marks is perceived as the true position of target. Then, the true positions will be coupled with the recorded time. Finally, the actual positions for each sampling time can be calculated by the interpolation method according to the detected positions and recorded time.

### 5.2. Experimental Analysis

**Figure 9:**
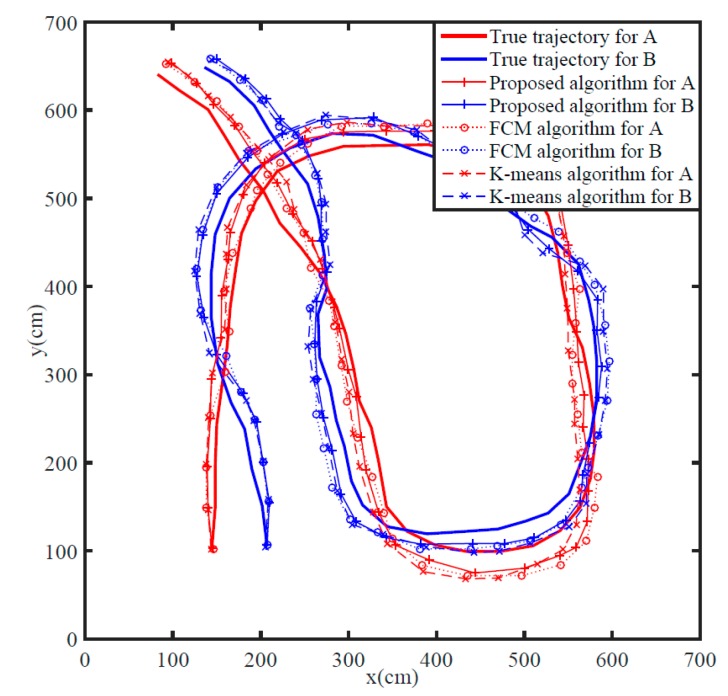
Tracking comparison among the algorithms.

Based on the above experimental setup, sequence data were collected from two closely moving persons and then used to estimate the target trajectory by the presented algorithm, K-means, and FCM methods separately, see Figure 9. 

**Figure 10:**
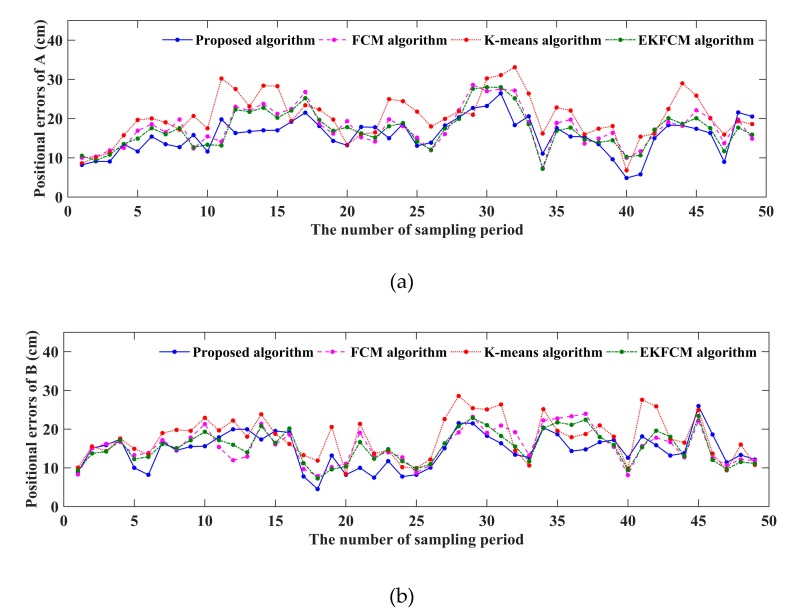
Positional errors for these the algorithms. (**a**) Positional errors with these algorithms for target A; (**b**) Positional errors with these algorithms for target B.

**Figure 11:**
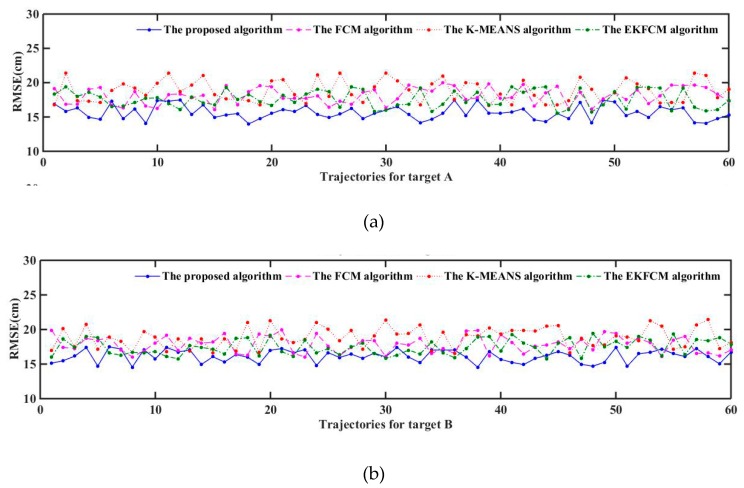
RMSE of 60 trajectories for targets A and B. (**a**) RMSE of these algorithms for Target A; (**b**) RMSE of these algorithms for Target B.

We counted the positional errors (PE) between the locations from each algorithm and truth locations, as shown in Figure 10, which shows the PE of the new algorithm, and found that they are basically less than that of other methods. In order to compare the performance of these algorithms more intuitively, RMSE was calculated and shown in Table 2. Besides, we have done 60 experiments and collected 60 trajectories which include the trajectory shown in Figure 9. We have calculated the RMSE of these trajectories and shown them in Figure 11. At last, we also calculate the MMSE for these trajectories, which is shown in Table 3. It demonstrates that MMSE of the new algorithm is still lower than that of others, which keeps consistent with the results of the simulation. Moreover, under Matlab R2014b with Intel(R) Core(TM) i5-4590 CPU at 3.3 GHz as well as 8 GB RAM, the average run time of tracking is 0.328s, which will satisfy the real-time requirement of our system.

## 6. Conclusions and Future Work

This paper mainly focuses on the classification problem among the sampling points of two adjacent targets. The proposed algorithm combined EKF and LS-SVM methods to obtain the best classification line for each sampling period, which was then devoted to precisely classify sampling points and calculate the observed centers of targets, as well as estimate the positions for closely moving targets based on EKF. Several simulations were conducted to demonstrate the feasibility and accuracy of the presented algorithm for tracking two closely moving targets in WSNs. Moreover, in contrast with state-of-the-art K-means, FCM and EKFCM methods, experimental and quantitative results further validate the efficiency and effectiveness of our proposal. In the near future, algorithm optimization and its extension to track more than two targets will be fully investigated. 

## Figures and Tables

**Table 1:** MMSE of the proposed and existing methods for two targets.

Target	K-Means	FCM	EKFCM	Proposed Method
A (cm)	15.7163	13.7165	13.7481	12.1711
B (cm)	15.8078	14.1137	13.4989	12.4182
Improvement rate (%)	21.99	11.65	9.75	-
Average Run Time (s)	0.1534	0.1674	0.1871	0.2162

**Table 2:** RMSE (cm) of proposed and existing methods for the experiment.

Target	K-Means	FCM	EKFCM	Proposed Method
A	20.9915	19.1967	17.8418	16.9961
B	18.2747	16.0483	15.9778	15.4728

**Table 3:** MMSE of proposed and existing methods for the experiment.

Target	K-Means	FCM	EKFCM	Proposed Method
A (cm)	18.8562	18.1433	17.6606	15.6904
B (cm)	18.9090	17.8874	17.4856	16.1786
Improvement rate (%)	15.61	11.54	9.32	-
